# HpaC Controls Substrate Specificity of the *Xanthomonas* Type III Secretion System

**DOI:** 10.1371/journal.ppat.1000094

**Published:** 2008-06-27

**Authors:** Christian Lorenz, Steve Schulz, Thomas Wolsch, Ombeline Rossier, Ulla Bonas, Daniela Büttner

**Affiliations:** Institut für Biologie, Bereich Genetik, Martin-Luther-Universität Halle-Wittenberg, Halle (Saale), Germany; The University of North Carolina at Chapel Hill, United States of America

## Abstract

The Gram-negative bacterial plant pathogen *Xanthomonas campestris* pv. *vesicatoria* employs a type III secretion (T3S) system to inject bacterial effector proteins into the host cell cytoplasm. One essential pathogenicity factor is HrpB2, which is secreted by the T3S system. We show that secretion of HrpB2 is suppressed by HpaC, which was previously identified as a T3S control protein. Since HpaC promotes secretion of translocon and effector proteins but inhibits secretion of HrpB2, HpaC presumably acts as a T3S substrate specificity switch protein. Protein–protein interaction studies revealed that HpaC interacts with HrpB2 and the C-terminal domain of HrcU, a conserved inner membrane component of the T3S system. However, no interaction was observed between HpaC and the full-length HrcU protein. Analysis of HpaC deletion derivatives revealed that the binding site for the C-terminal domain of HrcU is essential for HpaC function. This suggests that HpaC binding to the HrcU C terminus is key for the control of T3S. The C terminus of HrcU also provides a binding site for HrpB2; however, no interaction was observed with other T3S substrates including pilus, translocon and effector proteins. This is in contrast to HrcU homologs from animal pathogenic bacteria suggesting evolution of distinct mechanisms in plant and animal pathogenic bacteria for T3S substrate recognition.

## Author Summary

The Gram-negative plant pathogenic bacterium *Xanthomonas campestris* pv. *vesicatoria* is the causal agent of bacterial spot disease in pepper and tomato. Pathogenicity of *X. campestris* pv. *vesicatoria* depends on a type III protein secretion (T3S) system that injects bacterial effector proteins directly into the host cell cytosol. The T3S system is a highly complex nanomachine that spans both bacterial membranes and is associated with an extracellular pilus and a translocon that inserts into the host cell membrane. Given the architecture of the secretion apparatus, it is conceivable that pilus formation precedes effector protein secretion. The pilus presumably consists of two components, i.e., the major pilus subunit HrpE and HrpB2, which is required for pilus assembly. Secretion of HrpB2 is suppressed by HpaC that switches substrate specificity of the T3S system from secretion of HrpB2 to secretion of translocon and effector proteins. The substrate specificity switch depends on the cytoplasmic domain of HrcU, which is a conserved inner membrane protein of the T3S apparatus that interacts with HrpB2 and HpaC.

## Introduction

Many Gram-negative bacterial pathogens of plants and animals depend on a type III secretion (T3S) system to successfully infect their hosts [1]. The term “T3S system” refers to both translocation-associated and flagellar T3S systems that evolved from a common ancestor [2]. Eleven components of the membrane-spanning basal body are conserved, suggesting a similar overall architecture of the secretion apparatus [1],[3]. Main structural differences are found in the extracellular appendages associated with the basal body. The flagellar T3S apparatus is connected via an extracellular hook to the filament, the key bacterial motility organelle [4]. By contrast, the basal body of translocation-associated T3S systems is associated with an extracellular pilus (plant pathogens) or needle (animal pathogens), which serve as conduits for secreted proteins to the host-pathogen interface [1],[5]. Pilus and needle are proposed to be linked to the T3S translocon, a channel-like protein complex that is inserted into the eukaryotic plasma membrane and allows protein translocation into the host cell cytosol [6],[7].

Translocation-associated T3S systems secrete two types of proteins, i.e., extracellular components of the secretion apparatus such as needle/pilus and translocon proteins, and effectors that are translocated into the host cell [3]. Efficient secretion and/or translocation of T3S substrates depends on a signal in the N terminus, which is not conserved on the amino acid level [1],[8],[9]. In many cases, specific T3S chaperones bind to one or several homologous T3S substrates in the bacterial cytoplasm and promote stability and/or secretion of their respective binding partners. T3S chaperones are small, acidic and leucine-rich proteins that presumably guide secreted proteins to the secretion apparatus at the inner membrane [1],[10],[11].

Given the architecture of the T3S system, it is conceivable that secretion of extracellular components of the secretion apparatus precedes effector protein translocation. In translocation-associated and flagellar T3S systems from animal pathogenic bacteria, experimental evidence suggests that substrate specificity is altered by so-called T3S substrate specificity switch (T3S4) proteins, e.g., YscP from *Yersinia* spp. and the flagellar homolog FliK [12]–[14]. The substrate specificity switch depends on the C-terminal cytoplasmic domain of a conserved inner membrane protein of the FlhB/YscU family [12],[13]. T3S4 proteins are not highly conserved among different pathogens and have so far only been identified in animal pathogenic bacteria [14],[15]. It therefore remained enigmatic whether plant pathogenic bacteria employ similar mechanisms to orchestrate secretion of different T3S substrates.

In our laboratory, we study T3S of the plant pathogenic bacterium *Xanthomonas campestris* pv. *vesicatoria*, the causal agent of bacterial spot disease in pepper and tomato. The T3S system of *X. campestris* pv. *vesicatoria* is essential for bacterial growth and disease symptom formation in susceptible host plants and the induction of the hypersensitive response (HR) in resistant plants. The HR is a rapid programmed cell death at the infection site that is triggered upon recognition of individual effector proteins, also termed avirulence (Avr) proteins, in plants that carry a cognate disease resistance gene [16],[17]. In susceptible plants, effector proteins presumably modulate host cellular pathways to the pathogen's benefit and thus primarily act as virulence factors [18],[19].

The T3S system from *X. campestris* pv. *vesicatoria* is encoded by the 23-kb chromosomal *hrp* (hypersensitive response and pathogenicity) gene cluster, which is organized in eight operons and contains 25 genes [20]–[23]. Eleven genes (termed *hrc* for *hrp* conserved) encode proteins that are conserved among plant and animal pathogenic bacteria [24]. Most *hrp* genes are essential for bacterial pathogenicity [25]. Several Hrp proteins are secreted and thus constitute extracellular components of the T3S system such as the pilus protein HrpE and the translocon protein HrpF [25]–[27]. T3S of extracellular components of the secretion apparatus and effector proteins is presumably controlled by the products of *hpa* (*hrp-associated*) genes that are encoded in the *hrp* gene cluster and contribute to pathogenicity [28]–[31]. Examples are the export control protein HpaC, which is required for the efficient secretion of translocon and some effector proteins, and the global T3S chaperone HpaB, which promotes secretion and translocation of multiple effector proteins [30],[31].

In this study, we analyzed HrpB2, which is an essential pathogenicity factor of *X. campestris* pv. *vesicatoria*. HrpB2 is a 13.7-kDa protein that is encoded by the second gene of the *hrpB* operon and is secreted by the T3S system [25],[32]. Homologous proteins are present in *Xanthomonas* spp., *Burkholderia* spp. and *Ralstonia solanacearum*, suggesting that HrpB2 also plays an important role in other pathogens. In *X. campestris* pv. *vesicatoria*, HrpB2 is essential for pilus formation and T3S and is therefore presumably one of the first proteins that travel the T3S apparatus [25],[26]. However, the mode of HrpB2 action is unknown because HrpB2 does not share significant sequence or structural similarity with a protein of known function. Here, we provide experimental evidence that secretion of HrpB2 is required for bacterial pathogenicity. Secretion of HrpB2 is significantly enhanced in the absence of the export control protein HpaC. Protein-protein interaction studies showed that HrpB2 binds to HpaC and to the C-terminal domain of the conserved inner membrane protein HrcU, which also interacts with HpaC. Our data suggest that the interaction between HpaC and the C-terminal domain of HrcU promotes a switch in substrate specificity of the T3S system from HrpB2 secretion to secretion of translocon and effector proteins.

## Results

### HrpB2 is essential for bacterial pathogenicity and T3S

**Figure 1:**
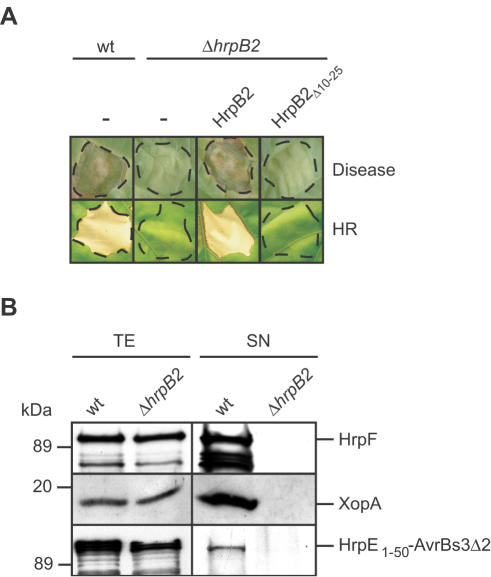
HrpB2 is essential for pathogenicity and T3S. (A) HrpB2 is crucial for disease symptom formation and HR induction. *X. campestris* pv. *vesicatoria* strains 85* (wt) and 85*Δ*hrpB2* (Δ*hrpB2*) carrying the empty vector (-) or synthesizing HrpB2 and HrpB2_Δ10–25_ as indicated were inoculated into susceptible ECW and resistant ECW-10R pepper plants. Dashed lines indicate the infiltrated areas. Disease symptoms and the HR were photographed five days after infiltration. (B) *In vitro* secretion of translocon and pilus proteins is abolished in the *hrpB2* deletion mutant. Strains 85* (wt) and 85*Δ*hrpB2* (Δ*hrpB2*) were incubated in secretion medium and total cell extracts (TE) and culture supernatants (SN) were analyzed by immunoblotting, using antibodies specific for HrpF, XopA and AvrBs3, respectively. HrpE_1–50_-AvrBs3Δ2 was expressed from an ectopic plasmid under control of the native *hrpE* promoter.

Previously, we identified HrpB2 as a T3S substrate of *X. campestris* pv. *vesicatoria* strain 85-10 [25]. Infection studies with *hrpB2* deletion mutant strains revealed that HrpB2 is crucial for disease symptoms in susceptible and the HR induction in resistant pepper plants [25]. Similar results were obtained with strains 85* and 85*Δ*hrpB2*, which carry hrpG*, a mutated version of the key regulatory gene *hrpG* in the bacterial chromosome (Fig 1A). hrpG* leads to constitutive expression of the T3S system and is key for the analysis of *in vitro* T3S [33]. It is noteworthy that *in planta* growth of hrpG* strains is like wild-type [34]. The *hrpB2* mutant phenotype could be complemented by ectopic expression of *hrpB2*, suggesting that loss of pathogenicity was specifically due to the deletion of *hrpB2* and did not result from a polar effect of the mutation on expression of other genes in the *hrpB* operon (Fig. 1A).

The fact that secretion of the effector protein AvrBs3 is abolished in *hrpB2* deletion mutants suggested that HrpB2 is involved in T3S [25]. To investigate the contribution of HrpB2 to secretion of additional T3S substrates, strains 85* and 85*Δ*hrpB2* were incubated in secretion medium, and total cell extracts and culture supernatants were analyzed by immunoblotting using specific polyclonal antibodies. We tested secretion of the putative translocon proteins HrpF and XopA, and the pilus protein HrpE. For technical reasons, HrpE was studied as a fusion protein consisting of the N-terminal 50 amino acids of HrpE and the reporter protein AvrBs3Δ2, which is a derivative of AvrBs3. AvrBs3Δ2 lacks the first 152 amino acids and thus the secretion and translocation signal [35]. It was previously demonstrated that the N-terminal 50 amino acids of HrpE restore secretion but not translocation of AvrBs3Δ2, indicating that they contain a functional T3S signal [36]. Fig. 1B shows that HrpF, XopA and HrpE_1–50_-AvrBs3Δ2 were present in the culture supernatant of the wild-type strain but were not detectable in the supernatant of the *hrpB2* deletion mutant, suggesting that HrpB2 is essential for secretion of these proteins.

### Secretion of HrpB2 is inhibited by HpaC

**Figure 2:**
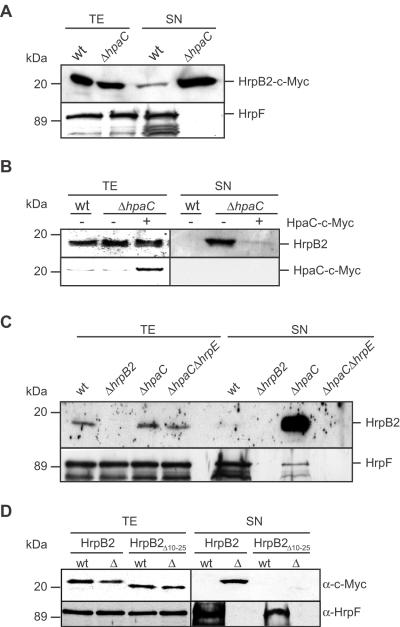
Secretion of HrpB2 is suppressed by HpaC. (A) Secretion of HrpB2-c-Myc is enhanced in *hpaC* deletion mutant strains. Strains 85* (wt) and 85*Δ*hpaC* (Δ*hpaC*) both synthesizing HrpB2-c-Myc were incubated in secretion medium. Total cell extracts (TE) and culture supernatants (SN) were analyzed by immunoblotting, using antibodies specific for the c-Myc epitope and HrpF, respectively. The blot was overexposed to visualize HrpB2-c-Myc in the culture supernatant of strain 85*. As expected, secretion of HrpF was reduced in the *hpaC* deletion mutant [31]. (B) The native HrpB2 protein is strongly secreted by strain 85*Δ*hpaC*. *In vitro* secretion assay with *X. campestris* pv. *vesicatoria* strains 85* (wt), 85*Δ*hpaC* (Δ*hpaC*) and 85*Δ*hpaC* expressing *hpaC-c-myc* from plasmid pDMhpaC as indicated. TE and SN were analyzed by immunoblotting, using HrpB2- and c-Myc-specific antibodies. (C) Secretion of HrpB2 is dependent on the T3S system. Strains 85* (wt), 85*Δ*hrpB2* (Δ*hrpB2*), 85*Δ*hpaC* (Δ*hpaC*) and 85*Δ*hpaC*Δ*hrpE* (Δ*hpaC*Δ*hrpE*) were incubated in secretion medium. TE and SN were analyzed by SDS-PAGE and immunoblotting, using HrpB2- and HrpF-specific antibodies. (D) The T3S signal of HrpB2 is located between amino acids 10 and 25. Strains 85* (wt) and 85*Δ*hpaC* (Δ) carrying expression constructs encoding HrpB2-c-Myc and HrpB2_Δ10–25_-c-Myc as indicated were incubated in secretion medium. TE and SN were analyzed by SDS-PAGE and immunoblotting, using c-Myc epitope- and HrpF-specific antibodies, respectively.

Since HrpB2 is secreted and is also required for T3S, it is presumably one of the first substrates that travel the T3S apparatus [25]. Notably, the amount of HrpB2 present in the culture supernatant of strain 85* is at the detection limit of the HrpB2-specific antibody [25]. Similar results were observed for a C-terminally c-Myc epitope-tagged version of HrpB2, suggesting that HrpB2 is only weakly secreted by the T3S system (Fig. 2A). To investigate whether HrpB2 secretion is regulated by the known export control proteins HpaB and HpaC, we performed *in vitro* T3S assays with strains 85*, the *hpaB* deletion mutant 85*Δ*hpaB* and the *hpaC* deletion mutant 85*Δ*hpaC*. We did not detect any influence of the global T3S chaperone HpaB on secretion of HrpB2. Interestingly, however, significantly increased amounts of HrpB2 were secreted by strain 85*Δ*hpaC* (Fig. 2). This was not due to a general increase of T3S in strain 85*Δ*hpaC* since secretion of the translocon protein HrpF was reduced when compared to the wild-type strain 85* (Fig. 2A and C). This is in agreement with the previous finding that HpaC is required for the efficient secretion of translocon and some effector proteins [31]. Oversecretion of HrpB2 in strain 85*Δ*hpaC* was specifically due to deletion of *hpaC* since the secretion phenotype was complemented by ectopic expression of *hpaC-c-myc* (Fig. 2B). We did not detect HrpB2 in the culture supernatant of the T3S double mutant 85*Δ*hpaC*Δ*hrpE*, which additionally lacks the Hrp pilus gene *hrpE* (Fig. 2C). We therefore conclude that increased HrpB2 secretion in strain 85*Δ*hpaC* was mediated by the translocation-associated T3S system.

### The N terminus of HrpB2 is crucial for protein function

Next, we investigated whether secretion of HrpB2 is crucial for protein function. For this, we analyzed N-terminal HrpB2 deletion derivatives. Surprisingly, deletion of the N-terminal 10 amino acids of HrpB2 did not abolish its secretion in wild-type and *hpaC* deletion mutant strains (data not shown). By contrast, secretion of a HrpB2 deletion derivative lacking amino acids 10 to 25 was severely reduced in strain 85*Δ*hpaC*, suggesting that amino acids 10 to 25 harbour at least part of the secretion signal (Fig. 2D). Notably, HrpB2_Δ10–25_ did not complement the *hrpB2* mutant phenotype with respect to disease symptom formation in susceptible and HR induction in resistant pepper plants (Fig. 1A). This was not due to the presence of the C-terminal c-Myc epitope since complementation studies were performed with untagged HrpB2 and derivatives. Immunoblot analysis of bacterial total cell extracts revealed that HrpB2_Δ10–25_ was stably synthesized in strain 85*Δ*hrpB2* (Fig. 2D). We therefore conclude that amino acids 10 to 25 are crucial for efficient secretion of HrpB2 and that HrpB2 secretion is presumably required for protein function.

### HrpB2 interacts with HpaC

**Figure 3:**
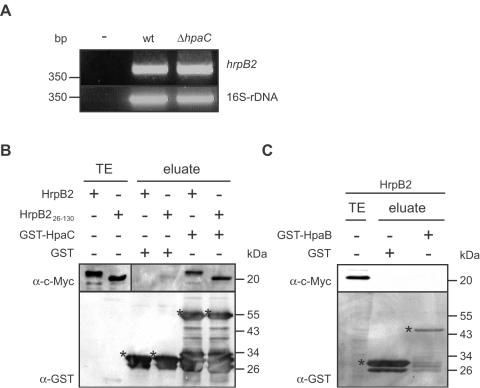
Suppression of HrpB2 secretion by HpaC is presumably due to protein-protein interactions. (A) The mRNA level of *hrpB2* is not affected by HpaC. RNA was isolated from strains 85* (wt) and 85*Δ*hpaC* (Δ*hpaC*) grown for 5 h in secretion-inducing medium. RT-PCR was performed using cDNA and primers specific for *hrpB2* and the 16S ribosomal DNA (16S-rDNA) that served as a constitutive control. RT-PCR amplicons were separated on a 2% agarose gel and stained with ethidium bromide. For a negative control (-), the template was replaced by water. (B) GST pull-down assays with HpaC and HrpB2 derivatives. GST and GST-HpaC were immobilized on glutathione sepharose and incubated with an *E. coli* lysate containing HrpB2-c-Myc and HrpB2_26–130_-c-Myc, respectively. The total cell lysate (TE) of *E. coli* expressing HrpB2-c-Myc and HrpB2_26–130_-c-Myc, respectively, and eluted proteins (eluate) were analyzed by SDS-PAGE and immunoblotting, using antibodies directed against the c-Myc epitope and GST, respectively. GST and GST fusion proteins are marked by asterisks, lower bands are degradation products. (C) The global T3S chaperone HpaB does not interact with HrpB2. GST and GST-HpaB were immobilized on glutathione sepharose and incubated with HrpB2-c-Myc. TE and eluate were analyzed as described in (B).

To investigate whether oversecretion of HrpB2 in the *hpaC* deletion mutant was due to increased *hrpB2* transcript levels, we performed reverse transcriptase (RT)-PCR analysis of strains 85* and 85*Δ*hpaC* grown under secretion-permissive conditions. Fig. 3A shows that *hrpB2* transcript levels were comparable in both strains, suggesting that deletion of *hpaC* did not affect the transcriptional regulation of *hrpB2*.

We therefore studied whether there is an interaction between HrpB2 and HpaC proteins using glutathione S-transferase (GST) pull-down assays. For this, GST and a GST-HpaC fusion protein were synthesized in *Escherichia coli*, immobilized on glutathione sepharose matrix and incubated with an *E. coli* lysate containing HrpB2-c-Myc. Bound proteins were eluted from the matrix and analyzed by immunoblotting using c-Myc epitope- and GST-specific antibodies. HrpB2-c-Myc specifically eluted with GST-HpaC but not with GST alone, indicating that HrpB2 interacts with HpaC (Fig. 3B). Similar results were obtained with an N-terminal HrpB2 deletion derivative that lacks the first 26 amino acids and thus at least part of the T3S signal (Fig. 3B; see above). The interaction between HpaC and HrpB2 is reminiscent of our previous finding that HpaC binds to different T3S substrates including translocon and effector proteins [31]. We did not observe an interaction between HrpB2 and the global T3S chaperone HpaB (Fig. 3C), which is in line with the fact that HpaB does not control HrpB2 secretion (see above).

### HrpB2 binds to the C-terminal domain of HrcU that is proteolytically cleaved

**Figure 4:**
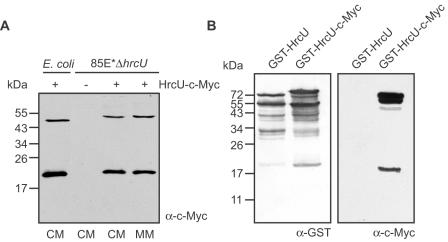
HrcU is cleaved in *E. coli* and *X. campestris* pv. *vesicatoria*. (A) Cleavage of HrcU-c-Myc. *E. coli* and *X. campestris* pv. *vesicatoria* strain 85E*Δ*hrcU* carrying the empty vector (-) or synthesizing HrcU-c-Myc as indicated were grown in complex (CM) or minimal medium (MM) as indicated. Total protein extracts were analyzed by immunoblotting, using a c-Myc-specific antibody. (B) Analysis of GST-HrcU fusion proteins. *E. coli* synthesizing GST-HrcU and GST-HrcU-c-Myc were grown in complex medium and total protein extracts were analyzed by immunoblotting, using a GST- and a c-Myc-specific antibody, respectively.

In animal pathogenic bacteria T3S substrate recognition is mediated by members of the conserved FlhB/YscU family of inner membrane proteins [37]–[39]. YscU, FlhB and their homologs contain four predicted transmembrane domains and a C-terminal cytoplasmic protein region that is cleaved between the asparagine and proline residues of the conserved NPTH motif [39]–[43]. To investigate a possible cleavage of the YscU/FlhB homolog HrcU from *X. campestris* pv. *vesicatoria*, we synthesized a C-terminally c-Myc epitope-tagged HrcU derivative in both *E. coli* and *X. campestris* pv. *vesicatoria* and analyzed protein extracts by immunoblotting using a c-Myc-specific antibody. We detected two proteins of approximately 50 kDa and 20 kDa in *E. coli* and *X. campestris* pv. *vesicatoria* extracts irrespective of the growth medium (Fig. 4A). Both proteins presumably correspond to full-length HrcU (39 kDa+5 kDa epitope tag) and the predicted C-terminal cleavage product (10 kDa+5 kDa epitope tag). The HrcU proteins migrate slower than predicted, which was previously also reported for other T3S system-associated proteins from *X. campestris* pv. *vesicatoria* [27],[29].

Because yeast two-hybrid-based interaction studies of proteins from *Xanthomonas axonopodis* pv. *citri* suggested an interaction between HrpB2 and the C-terminal domain of HrcU [44], we performed GST pull-down assays with HrpB2 and HrcU from *X. campestris* pv. *vesicatoria*. For this, we generated expression constructs encoding GST-HrcU, GST-HrcU-c-Myc and GST-HrcU_255–357_, the latter of corresponds to the C-terminal cytoplasmic domain of HrcU. To test for proteolytic cleavage, GST-HrcU and GST-HrcU-c-Myc were analyzed by immunoblotting of *E. coli* protein extracts, using GST- and c-Myc-specific antibodies. Both proteins and several degradation products were visualized by a GST-specific antibody (Fig. 4B). Furthermore, GST-HrcU-c-Myc and a smaller protein of approximately 20 kDa were also detected by a c-Myc specific antibody. The smaller protein presumably corresponds to the C-terminal cleavage product of HrcU (see Fig. 4A), indicating that GST-HrcU fusions are proteolytically cleaved (Fig. 4B).

**Figure 5:**
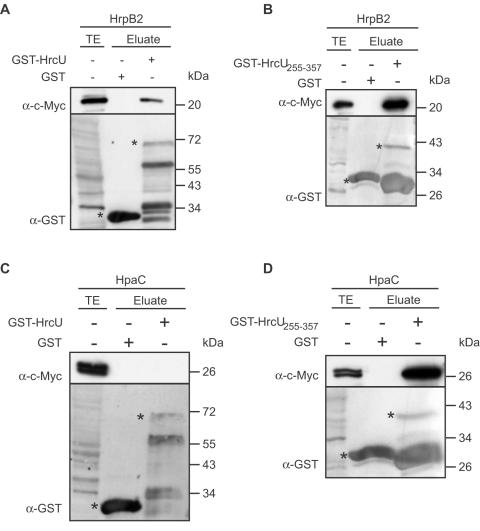
The C-terminal domain of HrcU interacts with HrpB2 and HpaC. (A) HrpB2 interacts with HrcU. GST and GST-HrcU were bound to glutathione sepharose and incubated with an *E. coli* lysate containing HrpB2-c-Myc. Total cell extract (TE) and eluates were analyzed by immunoblotting, using antibodies directed against the c-Myc epitope and GST, respectively. GST and GST fusion proteins are marked by asterisks, lower bands are degradation products. (B) HrpB2 binds to the C-terminal domain of HrcU. GST and GST-HrcU_255–357_ were immobilized on glutathione sepharose and incubated with HrpB2-c-Myc. TE and eluate were analyzed as described in (A). (C) Interaction studies with HpaC and HrcU. GST and GST-HrcU were immobilized on glutathione sepharose and incubated with an *E. coli* lysate containing HpaC-c-Myc. TE and eluate were analyzed as described in (A). (D) HpaC binds to the C-terminus of HrcU. GST and GST-HrcU_255–357_ were immobilized on glutathione sepharose and incubated with HpaC-c-Myc. TE and eluate were analyzed as described in (A).

For protein-protein interaction studies, GST-HrcU and GST-HrcU_255–357_ (HrcU C-terminal domain), immobilized on glutathione sepharose, were incubated with HrpB2-c-Myc. HrpB2-c-Myc eluted with GST-HrcU and GST-HrcU_255–357_, but not with GST alone, suggesting that HrpB2 interacts with the C-terminal domain of HrcU (Fig. 5A and B).

### HpaC interacts with the C-terminal domain of HrcU but not with the full-length protein

HrcU homologs from animal pathogenic bacteria are involved in the T3S substrate specificity switch [37],[39]. We therefore tested a possible interaction between HrcU and HpaC, which presumably acts as a T3S4 protein (see also below). When GST-HrcU was immobilized on glutathione sepharose and incubated with HpaC-c-Myc, we did not detect HpaC-c-Myc in the eluate (Fig. 5C). By contrast, HpaC-c-Myc coeluted with GST-HrcU_255–357_, suggesting that it interacts with the C-terminal domain of HrcU but not with the full-length protein (Fig. 5D). Since GST-HrcU is proteolytically cleaved (see Fig. 4B), we assume that the protein is correctly folded. Our data therefore suggest that the interaction between HpaC and HrcU depends on a certain conformation of the HrcU C terminus that is altered in the context of the full-length protein.

### The C-terminal domain of HrcU does not interact with HrpE, XopA and XopF1

**Figure 6:**
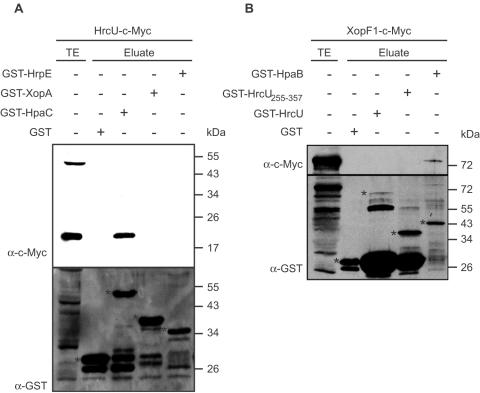
The C-terminal domain of HrcU does not interact with translocon and effector proteins. (A) GST, GST-HpaC, GST-XopA and GST-HrpE were immobilized on glutathione sepharose and incubated with HrcU-c-Myc. Total cell extract (TE) of *E. coli* synthesizing HrcU-c-Myc and eluates were analyzed by immunoblotting, using antibodies directed against the c-Myc epitope and GST, respectively. GST and GST fusion proteins are marked by asterisks, lower bands are degradation products. (B) GST, GST-HrcU, GST-HrcU_255–357_ and GST-HpaB were immobilized on glutathione sepharose and incubated with XopF1-c-Myc. TE and eluate were analyzed as described in (A).

Next, we investigated whether the C-terminal domain of HrcU also interacts with other secreted proteins, e.g., the putative translocon protein XopA, the pilus protein HrpE and the effector protein XopF1. For this, GST, GST-HpaC, GST-XopA and GST-HrpE were immobilized on glutathione sepharose and incubated with HrcU-c-Myc. Fig. 6A shows that the C-terminal cleavage product of HrcU (see above) was detected in the eluate of GST-HpaC but not of GST-XopA or GST-HrpE. This suggests that the C-terminal domain of HrcU interacts with HpaC but not with HrpE and XopA. We did not detect full-length HrcU-c-Myc in the eluate of GST-HpaC (Fig. 6A), which confirms our previous observation that HpaC specifically interacts with the C-terminal domain of HrcU but not with the full-length protein (see Fig. 5C and D).

To investigate a possible interaction between HrcU and the effector protein XopF1, we expressed XopF1 as a C-terminally c-Myc epitope-tagged derivative because a GST-XopF1 fusion protein was unstable in *E. coli*. XopF1-c-Myc was incubated with GST-HrcU, GST-HrcU_255–357_ and GST-HpaB, which was used as a positive control for the interaction assay. GST-HpaB was previously shown to interact with XopF1 [31]. As expected, XopF1-c-Myc was detected in the eluate of GST-HpaB but did not coelute with GST-HrcU and GST-HrcU_255–357_ (Fig. 6B). Taken together, our results suggest that the C-terminal domain of HrcU does not interact with the T3S substrates XopA, HrpE and XopF1. This is in contrast to the C-terminal region of the flagellar HrcU homolog FlhB, which interacts with several secreted proteins and is therefore presumably involved in substrate recognition [38].

### HpaC contains a putative T3S4 domain that is crucial for the interaction with the C-terminal domain of HrcU

The finding that HpaC is involved in control of T3S substrate specificity and interacts with the C-terminal domain of HrcU suggests that it acts similarly to T3S4 proteins that were identified in translocation-associated and flagellar T3S systems from animal pathogenic bacteria. Despite limited sequence conservation, known T3S4 proteins harbour a structurally conserved T3S4 domain in the C terminus, which is responsible for the substrate specificity switch [15],[45]. PSI-BLAST searches and hydrophobic cluster analysis showed that the T3S4 domain is not only present in proteins from animal pathogenic bacteria but also shares weak sequence similarity with the C terminus of HpaP from *Ralstonia solanacearum* [15]. HpaP is 27% sequence-identical to HpaC. A pairwise sequence alignment of HpaP and HpaC revealed that most conserved amino acids in the predicted T3S4 domain of HpaP are also present in HpaC or are substituted by amino acids with similar chemical properties (Fig. S1).

**Figure 7:**
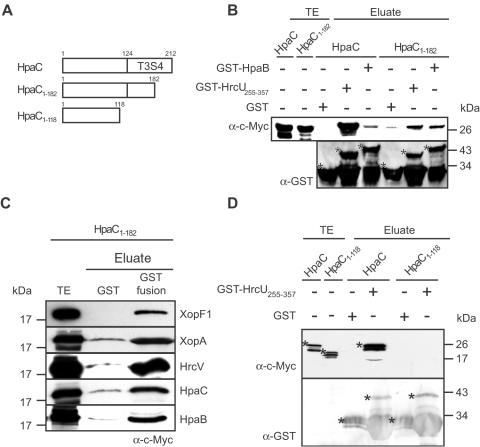
The C-terminal T3S4 domain of HpaC is crucial for the interaction with the C-terminal domain of HrcU. (A) Overview on HpaC deletion derivatives used in this study. The rectangle represents HpaC, the predicted T3S4 domain is indicated. Numbers refer to amino acid positions. (B) The C-terminal 30 amino acids of HpaC promote the interaction of HpaC with HrcU_255–357_. GST, GST-HrcU_255–357_ and GST-HpaB were immobilized on glutathione sepharose and incubated with HpaC-c-Myc and HpaC_1–182_-c-Myc as indicated. Total cell extracts (TE) of *E. coli* synthesizing HpaC-c-Myc and HpaC_1–182_-c-Myc, respectively, and eluates were analyzed by immunoblotting, using antibodies directed against the c-Myc epitope and GST, respectively. GST fusion proteins are marked by asterisks, lower bands are degradation products. (C) HpaC_1–182_-c-Myc interacts with known HpaC interaction partners. GST and GST fusion proteins containing XopF1, XopA, HrcV, HpaC and HpaB as indicated on the right side of the blots were immobilized on glutathione sepharose and incubated with HpaC_1–182_-c-Myc. TE and eluate were analyzed by immunoblotting, using a c-Myc-specific antibody. (D) The C terminal 94 amino acids of HpaC are crucial for the interaction with the C-terminal domain of HrcU. GST and GST-HrcU_255–357_ were bound to glutathione sepharose and incubated with HpaC-c-Myc and HpaC_1–118_-c-Myc, respectively. TE and eluate were analyzed as described in (B).

To investigate whether the predicted T3S4 domain of HpaC participates in the interaction with the C terminus of HrcU, we performed GST pull-down assays with C-terminal HpaC deletion derivatives, which are shown in Fig. 7A. HpaC_1–182_-c-Myc, which is deleted in the C-terminal 30 amino acids and thus lacks part of the predicted T3S4 domain, coeluted with GST-HrcU_255–357_, but not with GST alone (Fig. 7B). However, when compared to the full-length HpaC protein, which has a strong affinity for HrcU_255–357_, the interaction between HpaC_1–182_-c-Myc and GST-HrcU_255–357_ was significantly reduced (Fig. 7B). By contrast, binding of HpaC_1–182_-c-Myc to other known HpaC interaction partners such as HpaB, XopF1, XopA, HrcV and also the HpaC self-interaction was not affected (Fig. 7B and C) [31]. Next, we analyzed a HpaC deletion derivative, HpaC_1–118_-c-Myc, which lacks the C-terminal 94 amino acids and thus the complete T3S4 domain. The fact that HpaC_1–118_-c-Myc was not detectable in the eluate of GST-HrcU_255–357_ suggests that the predicted T3S4 domain of HpaC is important for the interaction with the C terminus of HrcU (Fig. 7D).

### The predicted T3S4 domain of HpaC is crucial for protein function

**Figure 8:**
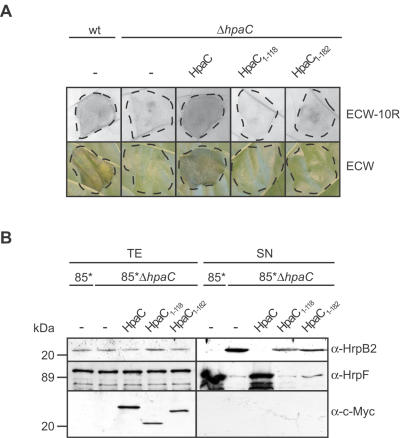
The C-terminal T3S4 domain of HpaC is crucial for protein function. (A) Complementation studies with HpaC derivatives. Strains 85-10 and 85-10Δ*hpaC* carrying the empty vector (-) or expression constructs encoding HpaC-c-Myc, HpaC_1–118_-c-Myc and HpaC_1–182_-c-Myc as indicated were inoculated into leaves of susceptible ECW and resistant ECW-10R pepper plants. For the better visualization of the HR, which was observed for strains 85-10 and 85-10Δ*hpaC* expressing *hpaC-c-myc*, leaves were bleached in ethanol two days after inoculation. Dashed lines indicate the infiltrated areas. (B) The C-terminal T3S4 domain of HpaC is crucial for T3S. *X. campestris* pv. *vesicatoria* strains 85* and 85*Δ*hpaC* carrying empty vector (-) or expression constructs encoding C-terminally c-Myc epitope-tagged derivatives of HpaC, HpaC_1–118_ and HpaC_1–182_ as indicated were incubated in secretion medium. Total cell extracts (TE) and culture supernatants (SN) were analyzed by SDS-PAGE and immunoblotting, using antibodies directed against HrpB2, HrpF and the c-Myc epitope, respectively.

To address whether the predicted T3S4 domain of HpaC is also important for protein function, we expressed HpaC and deletion derivatives in the *hpaC* deletion mutant. Fig. 8A shows that both HpaC_1–118_-c-Myc and HpaC_1–182_-c-Myc failed to complement the *hpaC* mutant phenotype with respect to (i) disease symptom formation and the HR induction in the plant, and (ii) oversecretion of HrpB2 *in vitro* (Fig. 8A and B). Furthermore, HpaC_1–118_-c-Myc and HpaC_1–182_-c-Myc did not restore the deficiency in HrpF secretion in strain 85*Δ*hpaC* (Fig. 8B). We therefore speculate that the T3S4 domain of HpaC and thus the interaction with the C-terminal domain of HrcU is essential for the HpaC-dependent substrate specificity switch.

## Discussion

In this study, we analyzed the pathogenicity factors HrpB2 and HpaC from *X. campestris* pv. *vesicatoria*. We discovered that HrpB2 is not only crucial for secretion of effectors but also of extracellular components of the secretion apparatus, i.e., the putative translocon proteins XopA and HrpF and the pilus protein HrpE. Since HrpB2 is itself secreted by the T3S system, it is presumably one of the first substrates that travels the secretion apparatus [25]. The analysis of N-terminal HrpB2 deletion derivatives revealed that the secretion signal of HrpB2 is located between amino acids 10 to 25 and is crucial for protein function. It is therefore possible that HrpB2 is an extracellular component of the secretion apparatus that promotes pilus assembly. However, HrpB2 is probably not a major pilus subunit since only low amounts of HrpB2 are secreted by the T3S system. Notably, the pilus protein HrpE is required for HrpB2 secretion and vice versa, suggesting that HrpB2 is not part of an extracellular needle-like structure below the pilus. An analogous finding was recently reported for the symbiotic bacterium *Rhizobium* strain NGR234. Pilus assembly and T3S in strain NGR234 depends on the secreted protein NopB that presumably associates with NopA, which is the major pilus subunit [46],[47].

The second important finding is that secretion of HrpB2 from *X. campestris* pv. *vesicatoria* is suppressed by the export control protein HpaC, which promotes secretion of translocon and effector proteins [31]. Proteins that differentially regulate secretion of different T3S substrates were described for flagellar or translocation-associated T3S systems and include, e.g., the flagellar chaperone FliS and T3S4 proteins from animal pathogenic bacteria [12]–[14],[48],[49]. We speculate that HpaC acts similarly to T3S4 proteins and alters the specificity of the secretion apparatus from early (HrpB2) to later (translocon and effector proteins) T3S substrates. We believe that this substrate specificity switch takes place at the protein level because HpaC interacts with HrpB2 and with the C-terminal domain of HrcU, which belongs to the FlhB/YscU family of inner membrane proteins [3],[12],[13]. This is in agreement with our previous finding that HpaC binds to different T3S substrates including translocon and effector proteins and also interacts with conserved inner membrane components of the T3S system such as HrcV [31]. It was therefore proposed that HpaC acts as a linker between secreted proteins and the secretion apparatus [31]. However, HpaC is dispensable for secretion of HrpB2. Targeting of HrpB2 to the secretion apparatus is presumably mediated by the C-terminal domain of HrcU, which interacts with both HrpB2 and HpaC. The latter interaction presumably depends on a certain conformation of the HrcU C terminus since we did not detect binding of HpaC to full-length HrcU.

The analysis of HpaC deletion derivatives revealed that the HrcU-binding site is located in the C terminus of HpaC, which contains the putative T3S4 domain [15] and is required for protein function. This observation suggests that the interaction between HpaC and the C-terminal domain of HrcU is required for HpaC-mediated suppression of HrpB2 secretion. Our data are reminiscent of the finding that the T3S4 protein FliK from *Salmonella* spp. interacts with the C-terminal domain of the HrcU homolog FlhB [37],[38]. It was proposed that binding of FliK induces a conformational change in the C–terminal cytoplasmic domain of FlhB and thus alters the substrate specificity of the flagellar T3S system from secretion of hook components to filament proteins [12],[50]. Since the C-terminal domain of FlhB interacts with several secreted proteins, it presumably serves as a docking point for T3S substrates [37],[38]. This clearly differs from the FlhB homolog HrcU from *X. campestris* pv. *vesicatoria* since the C-terminal domain of HrcU does not interact with translocon and effector proteins that were tested in this study. It is conceivable that T3S substrate binding in *X. campestris* pv. *vesicatoria* is mediated by other conserved inner membrane components of the T3S system such as HrcV or the putative ATPase HrcN [3].

The precise mechanism underlying the HpaC/HrcU-mediated substrate specificity switch in *X. campestris* pv. *vesicatoria* remains to be determined. We speculate that after activation of the T3S system, binding of HpaC to the C-terminal domain of HrcU inhibits the interaction between this domain and HrpB2 and thus blocks secretion of HrpB2. Preliminary GST pull-down assays revealed that HrpB2-c-Myc coelutes with GST-HrcU_255–357_ irrespective of the presence of HpaC, suggesting that all three proteins can form a complex. It still remains to be investigated whether both proteins simultaneously bind to the C-terminal domain of HrcU or whether they are both present in the eluate because they interact with each other.

Taken together, our data suggest that plant and animal pathogenic bacteria share similar mechanisms to switch the substrate specificity of the T3S system but that they differ in the components that recognize T3S substrates. Another important difference between plant and animal pathogenic bacteria concerns the length control of extracellular structures associated with the membrane-spanning secretion apparatus. In translocation-associated and flagellar T3S systems from animal pathogenic bacteria, the substrate specificity switch is coupled to length control of needle and hook structures. In the flagellar T3S system from *Salmonella* spp., for instance, FliK activates secretion of filament proteins after hook formation. Deletion of *fliK* leads to elongated hook structures, suggesting that FliK is required for hook length control [51]–[53]. Similarly to FliK, the T3S4 protein YscP from *Yersinia* spp. determines needle length in the translocation-associated T3S system [12]. Since YscP is itself secreted it was proposed that the N terminus of YscP anchors to the tip of the growing needle while the C terminus of the protein remains attached to the secretion apparatus and activates the substrate specificity switch [54]. According to this model, T3S4 proteins act as molecular rulers that are coupled to a substrate specificity switch [12],[15]. The molecular ruler model was challenged by the finding that the T3S4 protein InvJ from *Salmonella typhimurium* is required for formation of the inner rod of the T3S apparatus. It was suggested that formation of the inner rod triggers a conformational change in the secretion apparatus that leads to the substrate specificity switch [55]. This model is supported by the recent finding that the T3S4 protein YscP from *Yersinia* controls secretion of the predicted inner rod protein YscI [56]. Wood *et al.* identified YscI point mutants that allow effector secretion in the absence of a detectable needle structure, suggesting that the needle is not required for the substrate specificity switch.

The future challenge is to investigate the molecular mechanisms underlying the HpaC-mediated T3S substrate specificity switch in *X. campestris* pv. *vesicatoria*. Since HpaC is not secreted by the T3S system, it presumably does not act as a molecular ruler protein [31]. Electron microscopy studies have suggested that pilus length is not controlled by HpaC [26]. Furthermore, it should be emphasized that secretion of the Hrp pilus subunit HrpE is not affected in *hpaC* mutants [31]. In contrast to the relatively short (approximately 50 nm) T3S needle from animal pathogenic bacteria, the Hrp pilus from plant pathogens can reach a length of up to 2 µm that cannot be bridged by a single proteinaceous molecular ruler. We therefore speculate that HpaC acts as a T3S4 protein that is not involved in length control of extracellular structures of the T3S system. This hypothesis is supported by the fact that secretion-deficient derivatives of the T3S4 proteins YscP and FliK are still active, indicating that length control and substrate specificity switch functions can be uncoupled [54],[57].

## Materials and Methods

### Bacterial strains and growth conditions

Bacterial strains and plasmids used in this study are listed in Table 1. *E. coli* cells were cultivated at 37°C in lysogeny broth (LB). *X. campestris* pv. *vesicatoria* strains were grown at 30°C in NYG medium [58] or in minimal medium A [59] supplemented with sucrose (10 mM) and casamino acids (0.3%). Plasmids were introduced into *E. coli* by electroporation and into *X. campestris* pv. *vesicatoria* by conjugation, using pRK2013 as a helper plasmid in triparental matings [60]. For the generation of strain 85*Δ*hpaC*Δ*hrpE*, pOK-hrpEΔ9-93, which is a derivative of the suicide plasmid pOK1 (see Table 1), was introduced into the genome of *X. campestris* pv. *vesicatoria* strain 85*Δ*hpaC* by conjugation. Double cross-overs resulted in deletion mutants that were selected as described [28].

**Table 1:** Bacterial strains and plasmids used in this study.

	Relevant characteristics^a^	Reference or source
***X. campestris*** ** pv. ** ***vesicatoria***		
85-10	pepper-race 2; wild type; Rif^r^	[64]
85*	85-10 derivative containing the hrpG* mutation, which renders *hrp* gene expression constitutive	[34]
85*Δ*hrpB2*	nonpolar *hrpB2* deletion mutant of strain 85*	[25]
85*Δ*hpaC*	nonpolar *hpaC* deletion mutant of strain 85*	[31]
85-10Δ*hpaC*	nonpolar *hpaC* deletion mutant of strain 85-10	[31]
85*Δ*hpaB*	nonpolar *hpaB* deletion mutant of strain 85*	[30]
85*Δ*hpaB*Δ*hpaC*	*hpaB/hpaC* double mutant of strain 85*	[31]
85*Δ*hpaC*Δ*hrpF*	*hpaC/hrpF* double mutant of strain 85*	this study
85*Δ*hpaC*Δ*hrpE*	*hpaC/hrpE* double mutant of strain 85*	this study
85E*Δ*hrcU*	*hrcU* deletion mutant of strain 85E*	[26]
***E. coli***		
DH5α	F^−^ *recA hsdR17(r_k_^−^,m_k_^+^) Φ80dlacZ ΔM15*	Bethesda Research Laboratories, Bethesda, Md.
DH5α λpir	F^−^ *recA hsdR17(r_k_^−^,m_k_^+^) Φ80dlacZ ΔM15 [λpir]*	[65]
**Plasmids**		
pBlueskript(II) KS	phagemid, pUC derivative; Ap^r^	Stratagene
pUC119	ColE1 replicon; Ap^r^	[66]
pC3003	pUC19 containing a triple c-*myc* tag; Ap^r^	J. Kämper
pENTR/D TOPO	Gateway system donor vector; Km^r^	Invitrogen
pDSK602	broad-host-range vector; contains triple *lacUV5* promoter; Sp^r^	[67]
pGWB16	binary expression vector, contains *attR1-CmR-ccdB-attR2* upstream of 4×*c-myc* epitope-encoding sequence	[68]
pGEX-2TKM	GST expression vector; p*_tac_* GST *lacI* ^q^ pBR322	Stratagene
	*ori*; Ap^r^, derivative of pGEX-2TK with polylinker of pDSK604	[69]
pGEX-6P-1	GST expression vector	Stratagene
pOK1	suicide vector; *sacB sacQ mobRK2* oriR6K; Sm^r^	[28]
pOK-hrpEΔ9-93	pOK1 derivative containing *hrpE* with a stop codon insertion after codon 8	[26]
pRK2013	ColE1 replicon, TraRK^+^ Mob^+^; Km^r^	[60]
pL6HrpE_50_AvrBs3Δ2	pLAFR6 derivative encoding HrpE_1–50_-AvrBS3Δ2 under control of the *hrpE* promoter	[36]
pDMhpaB	pDSK604 derivative encoding HpaB-c-Myc	[30]
pDMhpaC	pDSK604 derivative encoding HpaC-c-Myc	[31]
pGhpaB	pGEX-2TKM expressing GST-HpaB	[31]
pGhpaC	pGEX-2TKM expressing GST-HpaC	[31]
pGxopA	pGEX-2TKM expressin GST-XopA	[30]
pGxopF1	pGEX-2TKM expressin GST-XopF1	[31]

a Ap, ampicillin; Km, kanamycin; Rif, rifampicin; Sp, spectinomycin; r, resistant.

Antibiotics were added to the media at the following final concentrations: ampicillin, 100 µg/ml; kanamycin, 25 µg/ml; rifampicin, 100 µg/ml; spectinomycin, 100 µg/ml; tetracycline, 10 µg/ml.

### Plant material and plant inoculations

The near-isogenic pepper cultivars Early Cal Wonder (ECW), ECW-10R and ECW-30R [61] were grown and inoculated with *X. campestris* pv. *vesicatoria* as described previously [22]. Bacteria were hand-infiltrated into the intercellular spaces of leaves at concentrations of 2×10^8^ cfu/ml in 1 mM MgCl_2_ if not stated otherwise. The appearance of disease symptoms and the HR were scored over a period of three to five days after inoculation. For better visualization of the HR, leaves were bleached in 70% ethanol.

### RNA analyses

For RT-PCR analysis, bacteria were grown in secretion medium. RNA extraction and cDNA synthesis were performed as described [62] and *hrpB2* transcripts were amplified by PCR. To exclude that RNA preparations contained genomic DNA, total RNA was used as a template in a control PCR using *hrpB2*-specific primers. The lack of detectable *hrpB2* amounts suggested that the RNA preparations were DNA-free (data not shown). Sequences of primers used in this study are available upon request.

### Generation of *hrpB2* expression constructs

For the generation of *hrpB2* expression constructs, *hrpB2* and N-terminal deletion derivatives were amplified by PCR from *X. campestris* pv. *vesicatoria* strain 85-10 and cloned into the *Eco*RI and *Hin*dIII sites of pDSK602. To create c-Myc epitope-tagged derivatives of HrpB2, *hrpB2* and truncated gene fragments were subcloned into the *Eco*RI/*Sac*I sites of pC3003, in frame with a triple-*c-myc* epitope-encoding sequence, and the resulting inserts were introduced into the *Eco*RI/*Hin*dIII sites of pDSK602. For the generation of expression constructs encoding HrpB2_Δ10–25_ and HrpB2_Δ10–25_-c-Myc, full-length *hrpB2* cloned into pUC119 or pC3003 was used as template for a PCR. PCR products were religated and the respective inserts were cloned into pDSK602.

### Generation of *hrcU* expression constructs

For the generation of GST fusion proteins, full-length *hrcU* and a fragment encoding amino acids 255 to 357, respectively, were amplified by PCR and cloned into the *Eco*RI/*Xho*I sites of pGEX-2TKM, respectively, downstream and in frame with the GST-encoding sequence. To construct a C-terminally c-Myc epitope-tagged HrcU derivative, *hrcU* was amplified by PCR, inserted into pENTR/D-TOPO and recombined into pDGW4M using Gateway technology (Invitrogen, Carlsbad, Calif.). pDGW4M is a Gateway-compatible derivative of pDSK602 containing *attR* sites, chloramphenicol resistance and *ccdB* genes and the 4× c-Myc-encoding sequence of vector pGWB16 inserted into the *Eco*RI/*Hin*dIII sites.

To generate a GST-HrcU-c-Myc expression construct, *hrcU* was amplified by PCR, subcloned by *Sac*I and partial *Eco*RI digest in pC3003, which contains a triple *c-myc*-encoding sequence and introduced into the *Eco*RI/*Sac*I sites of pGEX-6P-1, in frame with a *gst*-encoding sequence.

### Secretion experiments and protein analysis

*In vitro* secretion assays were performed as described [27]. Total cell extracts and culture supernatants were analyzed by SDS-PAGE and immunoblotting. We used polyclonal antibodies specific for HrpF [27], XopA [29], AvrBs3 [63] and HrpB2 [25], respectively, and monoclonal anti-c-Myc and anti-GST antibodies (Amersham Pharmacia Biotech, Freiburg, Germany). Horseradish peroxidase-labelled anti-rabbit, anti-mouse and anti-goat antibodies (Amersham Pharmacia Biotech) were used as secondary antibodies. Antibody reactions were visualized by enhanced chemiluminescence (Amersham Pharmacia Biotech). To ensure that no bacterial lysis had occurred, blots were routinely reacted with an antibody specific for the intracellular protein HrcN (data not shown) [25].

### GST pull-down assays

GST pull-down assays were performed as described previously [31]. Briefly, GST and GST fusion proteins were expressed in *E. coli* and bacterial cells from 50 ml cultures were broken with a French press. GST and GST fusions were immobilized on glutathione sepharose and incubated with a c-Myc epitope-tagged derivative of the putative interaction partner. Bound proteins were eluted with 10 mM reduced glutathione. 5 µl total protein lysates and 20 µl eluted proteins were analyzed by SDS-PAGE and immunoblotting. For the generation of GST-HrpE, *hrpE* was amplified by PCR and cloned into the *Eco*RI/*Xho*I sites of pGEX-2TKM. The same blot was always incubated with an anti-c-Myc and an anti-GST antibody, respectively.

## Supporting Information

Figure S1 The C terminus of HpaC contains a predicted T3S4 domain. Sequence alignment of HpaC from *X. campestris* pv. *vesicatoria* strain 85-10 (accession number CAJ22055) and HpaP from *R. solanacearum* (accession number CAB58249). Amino acid sequences were aligned using CLUSTAL W (http://www.ebi.ac.uk/clustalw/). Conserved amino acids are shaded black, similar amino acids are shaded grey. The black bar indicates the predicted T3S4 domain in HpaP, stars refer to amino acids that are conserved among HpaP and T3S4 domain-containing proteins [15].
(0.56 MB EPS)
